# Supplementary material for: Cavity-magnon-polariton spectroscopy of strongly hybridized electro-nuclear spin excitations in LiHoF4
Source: Sci Rep. 2024 Oct 24;14:25227. doi: 10.1038/s41598-024-75978-x (PMC11502850; doi:10.1038/s41598-024-75978-x)
Supplement: Supplementary file 1 — Supplementary Information. [file 41598_2024_75978_MOESM1_ESM.pdf]

Supplementary materials: Cavity-Magnon-Polariton spectroscopy of strongly hybridized electro-nuclear spin excitations in $LiHoF_4$

Yikai Yang[1,2], **Peter Babkevich**[2], **Richard Gaal**[2], **Ivica Živković**[2], **and Henrik M. Rønnow**[2,*]

[1]Department of Engineering Science, University of Oxford, Parks Road, Oxford OX1 3PJ, United Kingdom
[2]Laboratory of Quantum Magnetism. Institute of Physics, École polytechnique fédérale de Lausanne, Lausanne CH-1015, Switzerland
[*]henrik.ronnow@epfl.ch

Cavity field-Spin Interaction

The interaction between the cavity photon field and a magnetic sample is in essence Zeeman interaction:

$$\mathscr{H}^{int}_{cav-spin} = -\int_{V_s} \vec{\mu}\cdot\vec{B}(\mathbf{r})dr \tag{1}$$

$$= -\sum_c \int_{V_s} \sqrt{\frac{\mu_0\hbar\omega_c}{2}}(\hat{a}_c+\hat{a}_c^\dagger)\vec{\mu}\cdot\vec{B}'(\mathbf{r})dr \tag{2}$$

where $\vec{\alpha}$ stands for the magnetic moment and the integration is over the sample volume (V_s), and we used the usual quantization rule on cavity photon field[1]. For arbitrary shaped cavity, we normalize the field amplitude in the same fashion as in Ref.[2], thus the interaction Hamiltonian reads:

$$\begin{aligned}
\mathscr{H}^{int}_{cav-spin} &= -\sum_c \sqrt{\frac{\mu_0\hbar\omega_c}{2}}(\hat{a}_c+\hat{a}_c^\dagger)\frac{\int_{V_s}\vec{\mu}\cdot\vec{B}'(\mathbf{r})dr}{\sqrt{\int_{V_c}|\vec{B}'(\mathbf{r})|^2dr'}}\\
&= -\sum_c \sqrt{\frac{\mu_0\hbar\omega_c}{2}}(\hat{a}_c+\hat{a}_c^\dagger)\int_{V_s}dr\Big(\frac{1}{\sqrt{N_s}}\sum_{\mathbf{q}}\vec{\mu}_q e^{i\mathbf{q}\cdot\mathbf{r}}\Big)\cdot\int\frac{d^3\mathbf{k}}{(2\pi)^3}\frac{\vec{B}'(\mathbf{k})e^{i\mathbf{k}\cdot\mathbf{r}}}{\sqrt{\int_{V_c}|\vec{B}'(\mathbf{r}')|^2dr'}}\\
&= -\sum_c \sqrt{\frac{\mu_0\hbar\omega_c}{2}}\frac{\hat{a}_c+\hat{a}_c^\dagger}{\sqrt{\int_{V_c}|\vec{B}'(\mathbf{r}')|^2dr'}}\frac{1}{\sqrt{N_s}}\sum_{\mathbf{q}}\int\frac{d^3\mathbf{k}}{(2\pi)^3}\vec{\mu}_q\cdot\vec{B}'(\mathbf{k})\int_{V_s}e^{i(\mathbf{q}+\mathbf{k})\cdot\mathbf{r}}dr\\
&= -\sum_c \sqrt{\frac{\mu_0\hbar\omega_c}{2}}\frac{\hat{a}_c+\hat{a}_c^\dagger}{\sqrt{\int_{V_c}|\vec{B}'(\mathbf{r}')|^2dr'}}\frac{1}{\sqrt{N_s}}\sum_{\mathbf{q}}\int\frac{d^3\mathbf{k}}{(2\pi)^3}\vec{\mu}_q\cdot\vec{B}'(\mathbf{k})\frac{N_s}{V_s}\delta(\mathbf{q}+\mathbf{k})\\
&= -\sum_c \sqrt{\frac{\mu_0\hbar\omega_c\rho_s}{2}}(\hat{a}_c+\hat{a}_c^\dagger)\sum_{\mathbf{q}}|\vec{\mu}_q|\sqrt{\frac{[\int_{V_s}|\vec{B}'_\mu(\mathbf{r})|e^{i\mathbf{k}\cdot\mathbf{r}}dr]^2}{V_s\int_{V_c}|\vec{B}'(\mathbf{r}')|^2dr'}}
\end{aligned} \tag{3}$$

where in the second line we introduced Fourier transformations $\vec{\mu} = \frac{1}{\sqrt{N_s}}\sum_{\mathbf{q}}\vec{\mu}_q e^{i\mathbf{q}\cdot\mathbf{r}}$ and $\vec{B}'(\mathbf{r}) = \int\frac{d^3\mathbf{k}}{(2\pi)^2}\vec{B}'(\mathbf{k})\cdot e^{i\mathbf{k}\cdot\mathbf{r}}$, and again the total number of spins in the sample $N_s = V_s\rho_s$. In the last step, $\vec{B}'_\mu(\mathbf{r})$ is the magnetic component of the photon field projected along the sample spin polarization. As we haven't imposed any artificial restrains, the result should be in principle true for any cavity and lattice spin system.

Crystal Field Rotation

In $LiReF_4$, the R ions are located at a site with local $\bar{4}$ (S_4) local symmetry. The crystal field Hamiltonian takes the form,

$$\mathscr{H}_{\mathrm{CEF}} = \sum_{l=2,4,6} B_l^0\mathbf{O}_l^0 + \sum_{l=4,6} B_l^4\mathbf{O}_l^4 + B_l^{-4}\mathbf{O}_l^{-4}, \tag{4}$$

where Stevens operators $\mathbf{O}_l^4$ and $\mathbf{O}_l^{-4}$ are sometimes referred to as $\mathbf{O}_l^4(c)$ and $\mathbf{O}_l^4(s)$, respectively. In a coordinate system where z-axis is chosen to be along the four-fold axis, by symmetry we have 7 crystal field parameters B_l^m which are to be determined experimentally. A common approach in spectroscopic measurements such as by Hansen *et al.* [PRB **1**2, 5315 (1975)] is to chose a coordinate system where the x direction is chosen such that $B_4^{-4}=0$. Furthermore, one can approximate the $\bar{4}$ point symmetry by $\bar{4}2m$ (D_{2d}) symmetry, in which case $B_6^{-4}=0$. This greatly reduces the number of free parameters in the fit. The Stevens operators that we employ here are defined as,

$$\begin{aligned}
\mathbf{O}_2^0 &= 3\mathbf{J}_z^2 - X\mathbf{I} \\
\mathbf{O}_4^0 &= 35\mathbf{J}_z^4 - (30X-25)\mathbf{J}_z^2 + (3X^2-6X)\mathbf{I} \\
\mathbf{O}_4^4 &= (\mathbf{J}_+^4 + \mathbf{J}_-^4)/2 \\
\mathbf{O}_4^{-4} &= -\mathrm{i}(\mathbf{J}_+^4 - \mathbf{J}_-^4)/2 \\
\mathbf{O}_6^0 &= 231\mathbf{J}_z^6 - (315X-735)\mathbf{J}_z^4 \\
&\quad + (105X^2-525X+294)\mathbf{J}_z^2 \\
&\quad + (-5X^3+40X^2-60X)\mathbf{I} \\
\mathbf{O}_6^4 &= [(11\mathbf{J}_z^2 - X\mathbf{I} - 38\mathbf{I})(\mathbf{J}_+^4+\mathbf{J}_-^4) \\
&\quad + (\mathbf{J}_+^4+\mathbf{J}_-^4)(11\mathbf{J}_z^2 - X\mathbf{I} - 38\mathbf{I})]/4 \\
\mathbf{O}_6^{-4} &= -\mathrm{i}[(11\mathbf{J}_z^2 - X\mathbf{I} - 38\mathbf{I})(\mathbf{J}_+^4-\mathbf{J}_-^4) \\
&\quad + (\mathbf{J}_+^4-\mathbf{J}_-^4)(11\mathbf{J}_z^2 - X\mathbf{I} - 38\mathbf{I})]/4
\end{aligned}$$

where $X=J(J+1)$ and $\mathbf{I}$ is the identity matrix of size $(2J+1)\times(2J+1)$. One can see from the operators that in the case of $\bar{4}$ point symmetry, there will be imaginary terms in the Hamiltonian. In the case of $\bar{4}2m$ local symmetry, the total Hamiltonian can be made real.

For a general rotation $R(\psi,\mathbf{n})$ by an angle ψ about an axis in the direction of vector $\mathbf{n}=(\sin\theta\cos\phi,\sin\theta\sin\phi,\cos\theta)$, by considering a product of infinitesimal rotations, one finds the unitary transformation operator to be,

$$\mathbf{U}(R(\psi,\mathbf{n})) = \exp(-im\psi\mathbf{n}\cdot\mathbf{J}). \tag{5}$$

This result can be used to determine how the operators $\mathbf{J}$ transform under a finite rotation. We can apply the rotation operator on the Hamiltonian where $\mathscr{H}_{\mathrm{CEF}}^R = \mathbf{U}^\dagger \mathscr{H}_{\mathrm{CEF}}\mathbf{U}$. From the Stevens operators above, we find that $\mathbf{O}_l^0$ operators commute with $\exp(-im\psi\mathbf{J}_z)$ and the rotation operator has effectively no effect on these terms. However, $\mathbf{O}_l^{\pm4}$ terms contain combination of raising and lowering operators $\mathbf{J}_+^4 \pm \mathbf{J}_-^4$ resulting in transformation of the operators as $\{\mathbf{O}_6^4\} = \cos(4\psi)[\mathbf{O}_6^4] - \sin(4\psi)[\mathbf{O}_6^{-4}]$, etc. This reflects the more general rule that connects rotations of 3D space and their unitary representatives on the Hilbert space,

$$\mathbf{U}^\dagger(\psi,\mathbf{n})\mathbf{V}\mathbf{U}(\psi,\mathbf{n}) = R(\psi,\mathbf{n})\mathbf{V}. \tag{6}$$

An alternative way to tackle the problem for arbitrary rotation of the Stevens operators are done by use of the rotation of Racah operators and the fact that the Stevens operators are linear combinations of Racah operators. The Racah operators namely transform under rotations of the frame of coordinates as the spherical harmonics, whereas the Stevens operators transform as the tesseral harmonics. The Stevens operators, denoted $\mathbf{O}_l^m$, have the disadvantage of not having systematic transformation properties under rotations of the frame of coordinates. Another set of operators, the Racah operators, denoted $\tilde{\mathbf{O}}_{l,m}$, are tensor operators and they therefore have systematic transformation properties. The Racah and Stevens operators are related as,

$$\mathbf{O}_l^m \quad = \quad \frac{1}{\kappa_l^m}\left(\frac{2l+1}{8\pi}\right)^{1/2}\left[\tilde{\mathbf{O}}_{l,-m} + (-1)^m\tilde{\mathbf{O}}_{l,m}\right] \tag{7}$$

$$\mathbf{O}_l^0 \quad = \quad \frac{1}{\kappa_l^0}\left(\frac{2l+1}{4\pi}\right)^{1/2}\tilde{\mathbf{O}}_{l,0} \tag{8}$$

$$\mathbf{O}_l^{-m} \quad = \quad \frac{1}{\kappa_l^m}\left(\frac{2l+1}{8\pi}\right)^{1/2}\left[\tilde{\mathbf{O}}_{l,-m} - (-1)^m\tilde{\mathbf{O}}_{l,m}\right] \tag{9}$$

The normalising coefficients κ_l^m in tesseral harmonics are tabulated elsewhere in Danielsen and Lindgård, Risø Report No 259 (1972). A comprehensive analysis of transformation properties of Stevens operators for a general rotation (ϕ,θ) of the

frame of coordinates is described by Rudowicz [J. Phys. C: Solid State Phys. **18**, 1415 (1985)] and Mulak and Gajek, *The effective crystal field potential*, Elsevier Science Ltd, Oxford (2000). In the appendix Rudowicz gives a summary of a general rotation of Stevens operators. For the case where $\theta = 0$, we find that,

$$\{\mathbf{O}_6^4\} = \cos(4\phi)[\mathbf{O}_6^4] - \sin(4\phi)[\mathbf{O}_6^{-4}], \tag{10}$$

and similarly,

$$\{\mathbf{O}_4^4\} = \cos(4\phi)[\mathbf{O}_4^4] - \sin(4\phi)[\mathbf{O}_4^{-4}], \tag{11}$$

where the curly brackets denote a column matrix of Stevens operators in the original axis system and the square brackets denote the operator in the transformed axis system which is rotated by an azimuthal angle ϕ. The expressions for $\{\mathbf{O}_4^{-4}\}$ are obtained by replacing $\cos(m\phi) \Rightarrow \sin(m\phi)$ and $\sin(m\phi) \Rightarrow -\cos(m\phi)$. The transformation matrices $\mathbf{S}_l(\phi,\theta)$ defined,

$$\{\mathbf{O}_l\} = \mathbf{S}_l(\phi,\theta)[\mathbf{O}_l], \tag{12}$$

are real but are not orthogonal – their inverse matrices are not equal to the transposed ones.

For the special case given in Eq. 4, we find that under a rotation about z, the Stevens operators transform as,

$$\left\{ \begin{array}{c} \mathbf{O}_l^4 \\ \mathbf{O}_l^0 \\ \mathbf{O}_l^{-4} \end{array} \right\} = \begin{pmatrix} \cos(4\phi) & 0 & -\sin(4\phi) \\ 0 & 1 & 0 \\ \sin(4\phi) & 0 & \cos(4\phi) \end{pmatrix} \left[\begin{array}{c} \mathbf{O}_l^4 \\ \mathbf{O}_l^0 \\ \mathbf{O}_l^{-4} \end{array} \right], \tag{13}$$

or alternatively from the view point of crystal field parameters,

$$\left[\begin{array}{c} B_l^4 \\ B_l^0 \\ B_l^{-4} \end{array} \right] = \begin{pmatrix} \cos(4\phi) & 0 & \sin(4\phi) \\ 0 & 1 & 0 \\ -\sin(4\phi) & 0 & \cos(4\phi) \end{pmatrix} \left\{ \begin{array}{c} B_l^4 \\ B_l^0 \\ B_l^{-4} \end{array} \right\}. \tag{14}$$

Re-entrant Cavity Details

The microwave cavity used in the present work is a standard 3D re-entrant cavity with cylindrical symmetry[3]. The cavity is made from high purity copper, whose structure is illustrated in figure SS1(a). The cavity space is a cylinder of 24 mm in diameter and 5 mm in height, a changeable cylindrical re-entrant post is placed concentric to the cavity space. In order to tune the bare cavity resonant frequency between 2 GHz and 5 GHz, the heights of the re-entrant posts are varied so that the capacitance gap (between the top of the post and the lid of the cavity) ranges from 200 μm to 600 μm. The cavity is coupled to the measurement circuit using a single-loop-antenna inserted in the cavity at a location symmetric to the position of the sample.

To characterize the re-entrant cavity, we first use COMSOL Multiphysics to carry out finite element analysis for its eigen-mode. The resultant electrical and magnetic field (density) distribution is shown in, respectively, the upper and lower panels of figure S1(b) using the vertical cross section of the re-entrant cavity. The shaded area represents the copper walls and the white area represents the cavity space. In order to highlight the capacitance gap, the cross section isn't drawn to scale. We then proceed to measure the scattering parameter $|S_{11}|$ of an empty cavity when it is both critically and over- coupled to the measurement setup. We show an an example of the measurement result in figure S1(c). Note that the cavity resonant frequency shown in this example is slightly different from the one in the main text, but there is no fundamental difference between the two cavities. We also characterize the field dependence of the bare cavity resonant frequency through measured $|S_{11}|$ at low temperature (100 mK) and various high magnetic fields, the result of which is shown in figure S1(d). It is clear from fig.S1(d) that the field dependence of the resonant frequency is very small ($\sim$ 45 kHz/T) comparing to the dramatic shifts due to the $LiHoF_4$ sample in off-resonance measurement (figure 3).

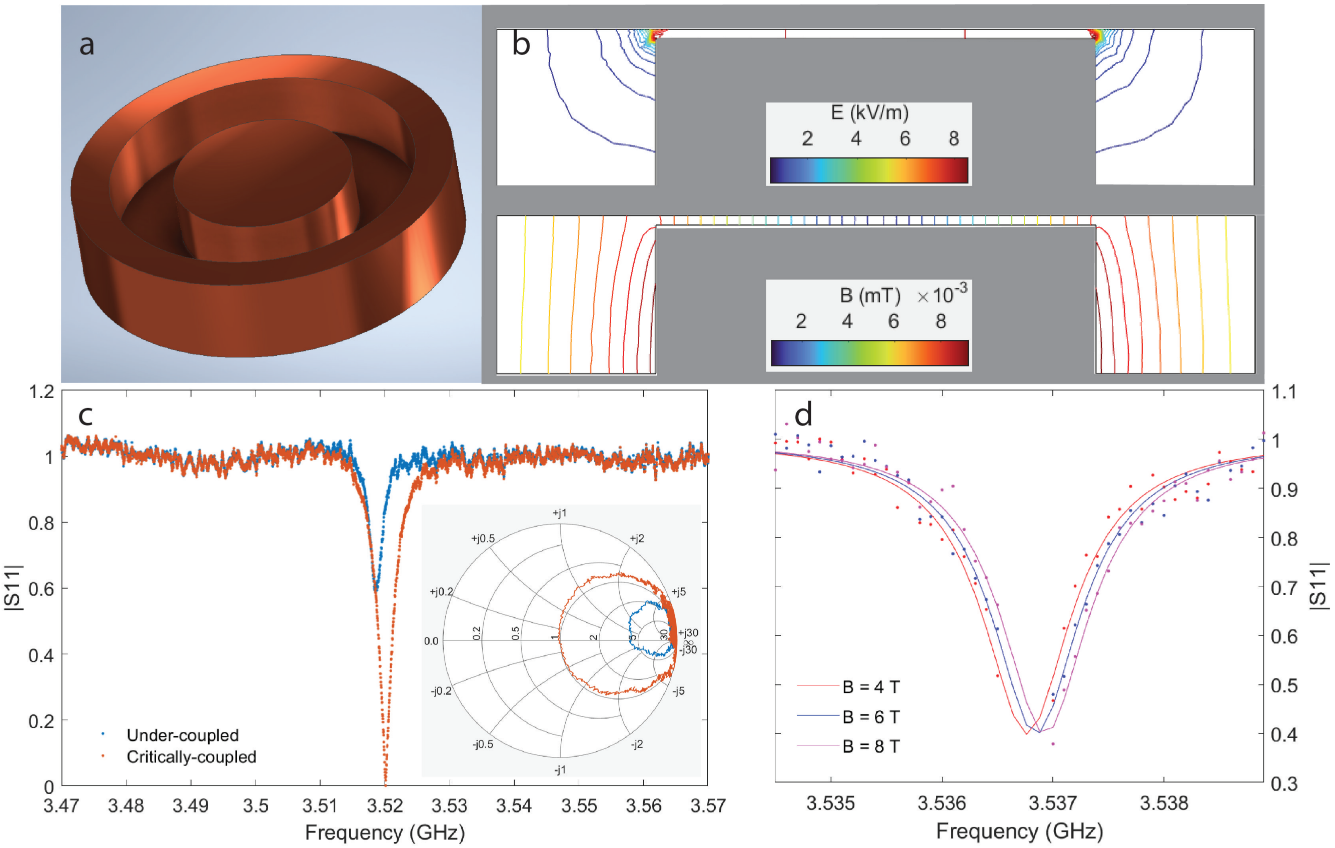

Figure S1. The re-entrant cavity used in the present work. (a) 3D illustration of the re-entrant cavity, mainly consists of a cylindrical cavity space and an re-entrant post located at the center of the cavity space. (b) Calculated electrical (upper panel) and magnetic (lower panel) field density distribution, shown in contours in the vertical (yz) cross section of the re-entrant cavity. (c) Experimentally measured $|S_{11}|$ at zero magnetic field and room temperature when the cavity is under- (blue) and critically (orange) coupled to the transmission line. Inset: the same measurement on smith chart. (d) Experimentally measured (dots) and fitted (solid lines) of $|S_{11}|$ at 100 mK and various magnetic fields.

References

1. Garrison, J. C. & Chiao, R. Y. *Quantum Optics* (Oxford University Press, 2008).
2. Flower, G., Goryachev, M., Bourhill, J. & Tobar, M. E. Experimental implementations of cavity-magnon systems: from ultra strong coupling to applications in precision measurement. *New J Phys* **21**, 095004 (2019). DOI 10.1088/1367-2630/ab3e1c.
3. Hamzah, H., Abduljabar, A., Lees, J. & Porch, A. A compact microwave microfluidic sensor using a re-entrant cavity. *Sensors* **910** (2018). DOI 10.3390/s18030910.
